# Periprosthetic Joint Infection by *Streptococcus bovis* Reveals Hidden Colorectal Cancer: A Case Report

**DOI:** 10.3390/life15091385

**Published:** 2025-09-01

**Authors:** George Viscopoleanu, Mihai-Sebastian Valeanu, Bogdan-Sorin Capitanu, Serban Dragosloveanu, Cristian Scheau

**Affiliations:** 1 Department of Orthopaedics, “Foisor” Clinical Hospital of Orthopaedics, Traumatology and Osteoarticular TB, 021382 Bucharest, Romania; george.viscopoleanu@gmail.com (G.V.); sebivaleanu@gmail.com (M.-S.V.); 2 Department of Orthopaedics and Traumatology, The “Carol Davila” University of Medicine and Pharmacy, 050474 Bucharest, Romania; 3 Department of Physiology, The “Carol Davila” University of Medicine and Pharmacy, 050474 Bucharest, Romania; cristian.scheau@umfcd.ro; 4 Department of Radiology and Medical Imaging, “Foisor” Clinical Hospital of Orthopaedics, Traumatology and Osteoarticular TB, 021382 Bucharest, Romania

**Keywords:** periprosthetic joint infection, *Streptococcus bovis*, colorectal cancer, hematogenous spread, translocation, case report

## Abstract

Periprosthetic joint infection (PJI) caused by *Streptococcus bovis* (*S. bovis*) is rare but clinically significant due to its established association with colorectal neoplasia. Early recognition and interdisciplinary management are essential to ensure favorable outcomes. We report the case of a 68-year-old woman who presented with a chronic fistula and signs of active infection 20 years after uncemented total hip arthroplasty. Cultures from the wound identified *S. bovis*, prompting further evaluation. Imaging and laboratory tests supported a diagnosis of chronic PJI. A two-stage revision was performed, beginning with implant removal, debridement, and placement of a vancomycin/gentamicin-loaded spacer. Given the pathogen’s known link to gastrointestinal malignancy, the patient underwent colonoscopy, which revealed a tubulovillous adenoma with carcinoma in situ. Surgical resection was performed with curative intent. Six months later, the patient underwent successful reimplantation. At three-month follow-up, clinical and radiographic assessments showed favorable recovery. This case reinforces the importance of gastrointestinal screening in patients with *S. bovis* PJI, as early detection of associated colorectal lesions may impact treatment strategies and prognosis.

## 1. Introduction

Periprosthetic joint infection (PJI) is a serious complication that can lead to revision arthroplasty and is associated with increased morbidity, mortality, and healthcare burden [1]. A synthesis of meta-analyses evaluating PJI incidence reported rates ranging from 0.34% to 2.45% in patients who had previously undergone total hip arthroplasty. The most commonly involved pathogens are *Staphylococcus aureus* and *Staphylococcus epidermidis* [2,3,4]. Streptococcus species are the second most frequently implicated, particularly in hematogenously acquired PJIs [5]. Among these *Streptococcus* species, the most frequently identified are *Streptococcus agalactiae* and *Streptococcus dysgalactiae*, whereas *viridans* group streptococci and *Streptococcus pneumoniae* are less commonly encountered [6].

The *Streptococcus bovis* (*S. bovis*) group consists of Gram-positive cocci that are part of the normal gastrointestinal flora and are known opportunistic pathogens implicated in endocarditis, bacteremia, and PJIs [7]. Although PJIs caused by *S. bovis* are uncommon, they have a well-established association with occult colorectal neoplasia, similar to the link observed in *S. bovis* endocarditis [8,9]. Recent studies have reinforced this association, for instance, Thompson et al. reported that in a series of nine *S. bovis* PJIs, 5 out of 7 patients who underwent colonoscopy were found to have colonic polyps or malignancy, including one case of colorectal carcinoma [10].

The proposed mechanism involves bacterial translocation through compromised intestinal mucosa, followed by hematogenous spread and seeding of prosthetic material [11]. Given this correlation, the current literature emphasizes the critical importance of gastrointestinal evaluation in patients with *S. bovis* orthopedic infections. Colonoscopy is recommended for all patients with *S. bovis* PJIs to promptly identify and treat any underlying colorectal cancer or advanced polyp lesions [12].

While the association between *S. bovis* bacteremia and colorectal cancer is well established, there is limited literature specifically addressing this pathogen in the context of PJI. This case report aims to illustrate and share our clinical experience in diagnosing and managing such infections, highlighting the diagnostic challenges and therapeutic considerations involved.

## 2. Case Report

**Figure 1:**
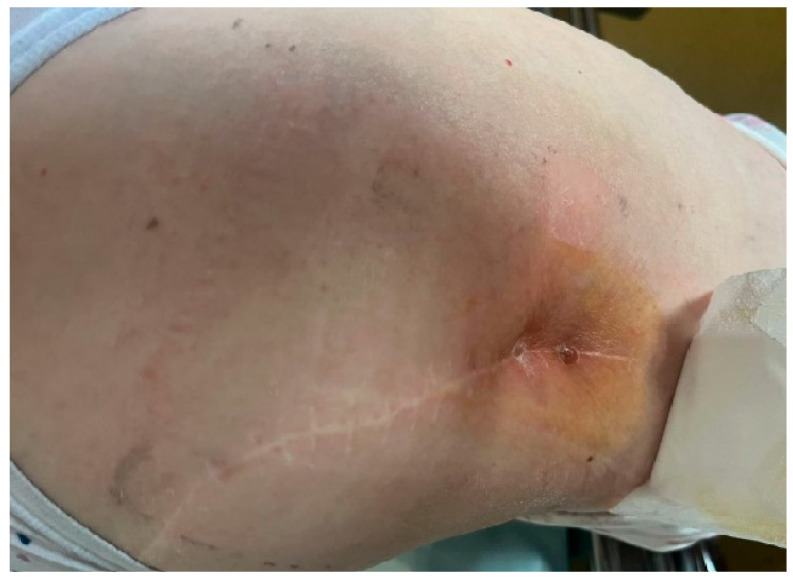
Clinical presentation at initial evaluation: active fistula located in the distal third of the postoperative scar, with visible signs of inflammation, local induration, and surrounding erythema.

This case involves a 68-year-old caucasian female who presented to our outpatient clinic with an active fistula located in the distal third of the postoperative scar on the posterolateral aspect of the thigh, accompanied by classical Celsian signs (erythema, warmth, swelling, and tenderness) (Figure 1). Her medical history was notable for a total uncemented hip arthroplasty performed 20 years earlier for hip arthritis.

At the time of presentation, the patient was in good general condition, afebrile, and had a normal range of motion in the affected limb. Past medical history included dyslipidemia, grade I primary hypertension, and class I obesity.

Symptoms began approximately five months prior to presentation, when the patient noted the development of a fistula with purulent discharge at the site of the previous surgical incision. Two months after symptom onset, she sought medical care at another orthopedic department, where she underwent local debridement, wound irrigation, and dressing changes. She was prescribed oral antibiotic therapy with Augmentin 1 g every 12 h for one month.

Despite this treatment, the symptoms persisted. Two months later, the patient consulted a different physician, who obtained a wound secretion sample for microbiological analysis. Culture results were positive for *S. bovis*, which showed sensitivity to clindamycin, linezolid, levofloxacin, penicillin, and vancomycin, based on antibiotic susceptibility testing. The patient was prescribed a three-week course of oral trimethoprim–sulfamethoxazole and was advised to begin physical therapy.

One month later, due to lack of clinical improvement, the patient presented to our clinic, where we decided to admit her for further investigation and appropriate surgical management.

**Figure 2:**
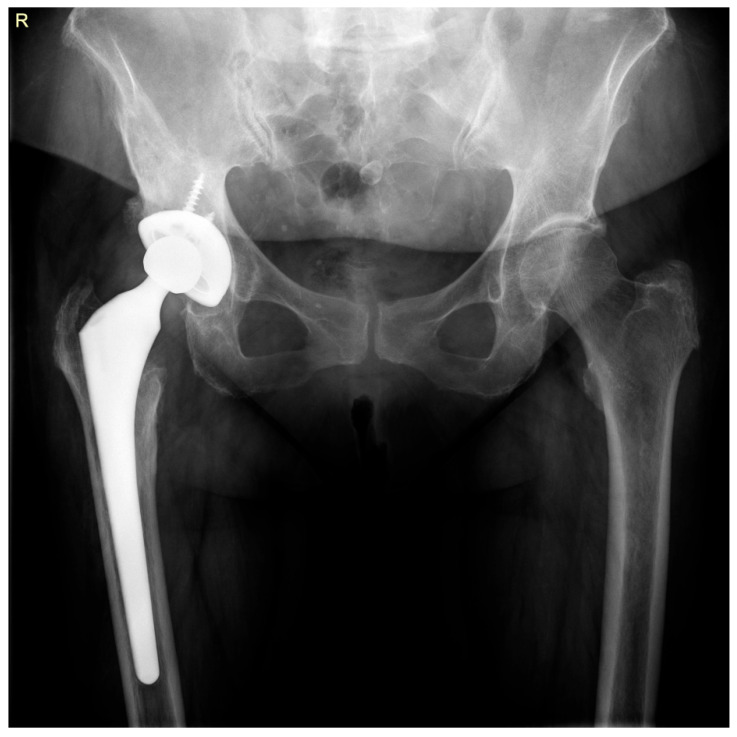
Radiographic findings at presentation: right hip prosthesis with well-positioned components, minimal polyethylene wear, and signs of osteolysis in Gruen zones 1 and 2 of the femur.

Upon admission, standard radiographs were obtained (Figure 2). Additionally, blood samples were collected for complete blood count, inflammatory markers, and coagulation profile, and a urine sample was obtained for culture.

Laboratory results revealed a mild anemia, with a hemoglobin (Hb) level of 11.6 g/dL (normal range: 12.0–16.0 g/dL), and signs of systemic inflammation, including an elevated erythrocyte sedimentation rate (ESR) of 58 mm/h (normal range: 0–30 mm/h), a C-reactive protein (CRP) level of 2.3 mg/dL (reference limit: <0.3 mg/dL), and an increased fibrinogen level of 496 mg/dL (normal range: 180–350 mg/dL). The urine culture was negative.

Based on the cumulative clinical, microbiological, and imaging findings, a diagnosis of PJI caused by *S. bovis* was established. As a result, a two-stage revision procedure was planned.

**Figure 3:**
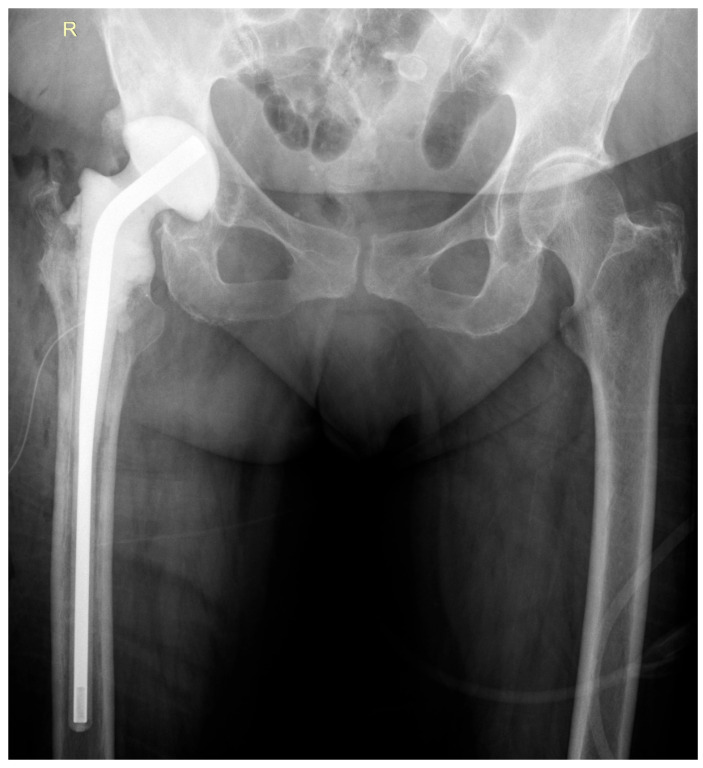
Postoperative anteroposterior (AP) view of the pelvis demonstrating the antibiotic-loaded spacer in appropriate position.

During the first-stage surgery, the infected prosthesis was explanted, followed by extensive surgical debridement and fistulectomy. A preformed, antibiotic-loaded spacer—Vancogenx^®^-Space Hip spacer (Tecres S.p.A., Sommacampagna, Italy)—was implanted (Figure 3). Intraoperative assessment indicated a low-grade infection, with mildly affected surrounding soft tissues and partially loosened prosthetic components. Intraoperative samples were collected for bacterial culture and microbiological analysis.

The postoperative course was uneventful. The patient remained afebrile, with favorable local and general evolution. The results from the intraoperative samples confirmed the findings of previous tests, identifying *S. bovis* with susceptibility to the majority of antibiotics tested (penicillin, cefuroxime, ceftriaxone, clindamycin). The surgical wound showed good healing, with no evidence of secretion or signs of reinfection. During hospitalization, she received intravenous antibiotic therapy consisting of vancomycin 1 g every 12 h and ceftriaxone 2 g daily for two weeks. Inflammatory markers demonstrated a descending trend, indicating resolution of the infectious process.

**Figure 4:**
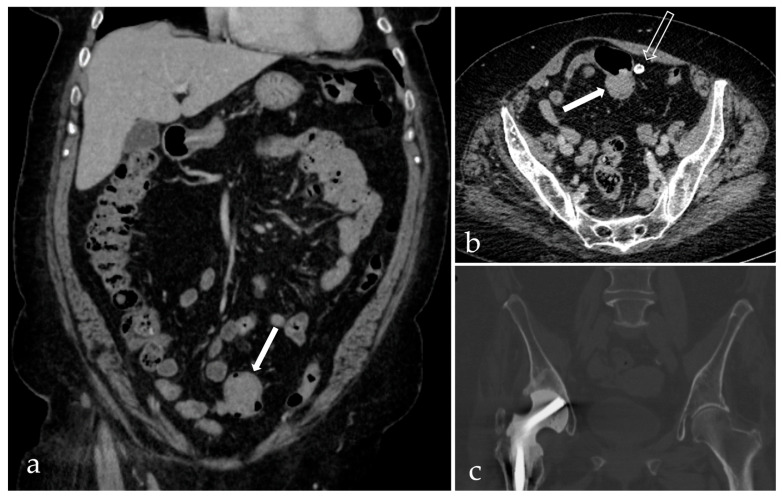
Computed tomography scan: portal venous phase coronal reformat (**a**) and axial image (**b**) depicting an enhancing polypoid tumor of the sigmoid colon (arrow); pericolonic ring-like calcification (empty arrow). Coronal reformat showing the spacer implanted in the right hip (**c**).

Given the identified pathogen and its known association with bacterial translocation and PJI, as supported by the literature, the patient was transferred to a specialized infectious disease hospital. The goal was to complete intravenous antibiotic therapy and to perform further investigations aimed at identifying potential colorectal pathology. A contrast-enhanced thoracoabdominal-pelvic CT scan revealed a tumor-like mass measuring approximately 23 × 48 mm in the sigmoid colon (Figure 4).

**Figure 5:**
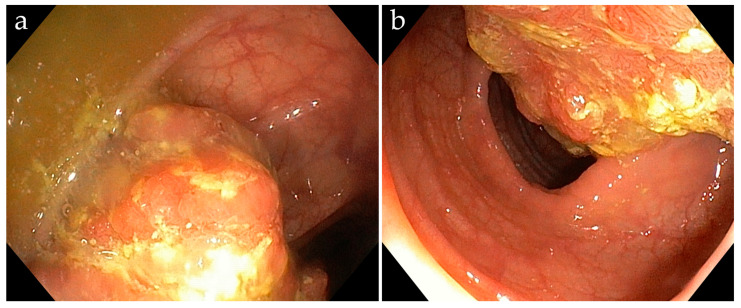
Macroscopic appearance of the tumor observed during colonoscopy: a friable, semi-circumferential, ulcerated lesion measuring approximately 5 cm (**a**,**b**).

Subsequent colonoscopy confirmed a friable, semi-circumferential, ulcerated soft tissue lesion approximately 5 cm in size, located 35 cm from the anal verge (Figure 5). Multiple biopsies were taken from the lesion.

Histopathological examination of the resected specimen identified a tubular adenocarcinoma in situ (Haggitt level 0) arising within a tubulovillous adenoma exhibiting high-grade dysplasia. The tumor was staged as pTisN0MxL0V0R0, indicating carcinoma in situ with no regional lymph node involvement, indeterminate distant metastasis, and absence of lymphatic or vascular invasion or residual tumor.

The gastroenterologist referred the patient to the general surgery department for curative management. A segmental sigmoid resection of approximately 15 cm, including adjacent adipose tissue, was performed, with restoration of bowel continuity. Based on the histopathological findings, no additional oncological treatment was indicated, and the malignancy was considered fully resected. The postoperative evolution was favorable.

Six months later, the patient returned to our department for the second stage of the surgical protocol. At the time of admission, inflammatory markers were within normal or near-normal limits: erythrocyte sedimentation rate (ESR) was 29 mm/h (normal range: 0–30 mm/h), C-reactive protein (CRP) was 0.5 mg/dL (reference limit: <0.3 mg/dL), and fibrinogen level was 422 mg/dL (normal range: 180–350 mg/dL).

**Figure 6:**
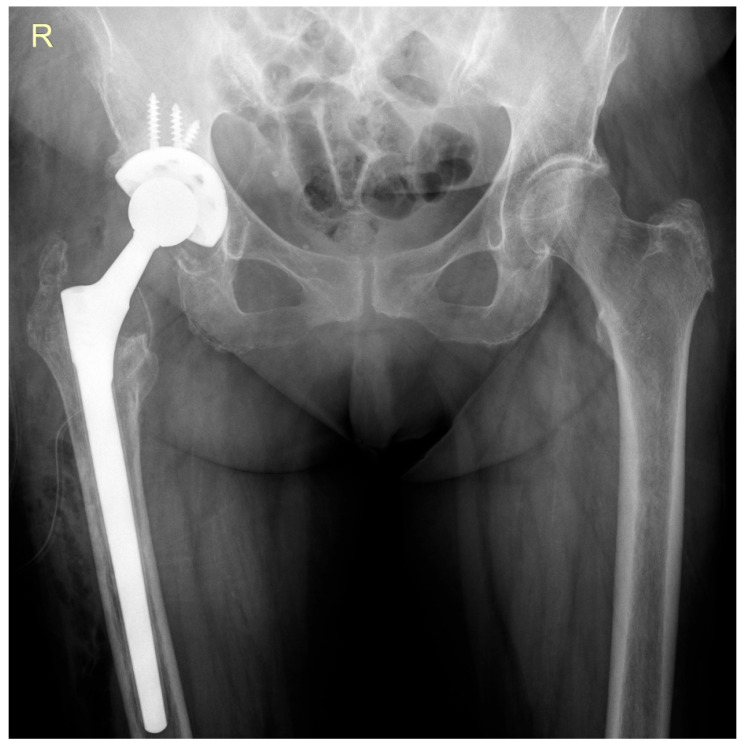
Follow-up anteroposterior pelvic radiograph showing the revision prosthesis in satisfactory position.

The surgical procedure involved removal of the antibiotic-loaded spacer and performance of a hip revision arthroplasty using a Zimmer Wagner SL^®^ femoral stem (Zimmer Biomet, Warsaw, IN, USA) and a Zimmer Trilogy IT^®^ acetabular component (Zimmer Biomet, Warsaw, IN, USA) (Figure 6). Intraoperative tissue samples were collected for microbiological analysis.

Postoperatively, the patient received intravenous antibiotic therapy consisting of vancomycin 1 g every 12 h and ceftriaxone 2 g daily for five days. All intraoperative cultures remained negative for bacterial growth during the 14-day observation period. The patient was discharged in good general condition—afebrile, with favorable local wound healing and a descending trend in inflammatory markers.

Upon discharge, she was prescribed oral antibiotic therapy with rifampicin 600 mg per day and cefuroxime 500 mg every 12 h, for a duration of 10 days. She was advised to use a walker for six weeks with gradual progression to full weight-bearing. At the most recent follow-up visit, three months postoperatively, both radiological and clinical evaluations demonstrated favorable outcomes, with no signs of reinfection or mechanical complications.

## 3. Discussion

This case clearly supports the association between colorectal pathology and *S. bovis* bacteremia, which can lead to PJI. Although PJIs caused by this pathogen are rare, they are clinically significant due to the well-documented link between this pathogen and underlying colorectal neoplasia [13,14,15]. Our case highlights this connection, as the identification of *S. bovis* in the PJI prompted further gastrointestinal evaluation, ultimately revealing a high-grade dysplastic adenoma with carcinoma in situ in the sigmoid colon.

This finding is consistent with previous studies that draw attention to the importance of colonoscopic screening in patients presenting with *S. bovis* infections outside the gastrointestinal tract [10,16,17,18,19,20]. Early detection and treatment of colorectal lesions can significantly improve patient outcomes and prevent further complications. The two-stage exchange protocol combined with targeted antibiotic therapy was effective in managing the PJI, while surgical resection of the colorectal tumor achieved curative intent.

Even though *Staphylococcus* species are the most frequently implicated in hematogenously acquired PJIs, *Streptococcus* species represent the second most common bacterial group, accounting for approximately 10–20% of cases in large cohort studies. The literature remains debatable regarding their comparative clinical course and outcomes, with some reports suggesting better response rates to debridement and antibiotic therapy compared with *Staphylococcus aureus*, while others indicate similar relapse risks, particularly when prosthetic retention is attempted [21,22].

*S. bovis* (now reclassified within the *Streptococcus gallolyticus* group) is part of the normal intestinal flora in humans. Although typically a commensal organism, it can become opportunistic under certain conditions [7,23]. There is a well-established association between *S. bovis* bacteremia and colorectal cancer or advanced colonic lesions. The bacterium demonstrates pro-inflammatory activity, stimulating the production of cytokines, free radicals, and nitric oxide, all of which contribute to tumorigenesis. Due to this connection, colonoscopy is strongly recommended whenever *S. bovis* is isolated from blood cultures or infected tissue, to rule out underlying colorectal pathology [18,24,25]. The proposed mechanism for PJI involves bacterial translocation across a compromised intestinal barrier, leading to bacteremia and hematogenous seeding of prosthetic surfaces. Once at the implant site, the bacteria may form a biofilm, enabling persistent infection and resistance to host immune responses and antibiotics [19,20,26].

Bacteremia caused by *S. bovis* is associated not only with colorectal cancer but also with premalignant colonic lesions. For example, in a case report by González et al., a patient who had undergone total knee arthroplasty developed a PJI with *S. bovis* three years postoperatively. The patient was referred for colonoscopy, which revealed a tubulovillous adenoma with low-grade dysplasia [16]. Furthermore, Thompson et al. reported a series of nine PJI cases involving *S. bovis*. Among the patients who underwent colonoscopy, five were found to have colonic polyps (including one carcinoma and two dysplastic lesions), while two others had abnormal findings of the intestinal mucosa [10]. These studies reinforce the importance of early and appropriate investigation, including colonoscopy, in patients with *S. bovis* infections, as such evaluations can lead to the identification of precancerous or malignant lesions that might otherwise progress undetected.

In our case, the two-stage hip revision approach was selected due to the well-established association between *S. bovis* and underlying colorectal pathology, implying that the source of infection remained active until properly diagnosed and treated, as well as the fact that the clinical criteria for a one-stage revision were not met [27,28,29]. The patient presented with a chronic fistula and clear signs of active infection, which contraindicated a single-stage procedure. The use of an antibiotic-loaded hip spacer containing vancomycin and gentamicin was deemed appropriate, as the isolated bacterial strain was susceptible to both agents. Moreover, the spacer allowed the patient to maintain a degree of mobility and provided the necessary time to undergo appropriate evaluation and treatment of the underlying colorectal disease, facilitating safe progression to the second stage of the revision surgery.

A key particularity of this case lies in the delayed surgical intervention and initial therapeutic approach. The patient was managed conservatively with antibiotic therapy alone, without a full diagnostic workup to determine the underlying cause of the fistula and infection. While this approach provided temporary infection control, it carried significant risk: progressive septic degradation of the prosthesis and most importantly, delayed recognition of a serious underlying condition. Ultimately, colorectal cancer was discovered only later, but still at the in situ stage, which allowed curative resection and subsequent hip revision surgery under favorable clinical circumstances. Given these aspects, the key point of discussion is how the infectious presentation and the malignant condition interacted within the diagnostic process, raising the question of a possible relationship between the two. There is no direct pathophysiological relationship between the hip fistula due to periprosthetic infection and the patient’s colorectal cancer. However, the coexistence of these two conditions is clinically significant. The chronic fistula, attributed to prosthetic infection, diverted attention away from a more complete diagnostic evaluation and thus masked the presence of the malignancy. This case highlights how complex infections can obscure coexisting pathologies, reinforcing the importance of thorough systemic evaluation in patients with atypical or persistent presentations.

This case report has several limitations. First, the follow-up period is relatively short, limiting our ability to assess long-term outcomes such as late reinfection, implant survival, or functional recovery. Ongoing monitoring will be necessary to draw more definitive conclusions. Second, the case was managed in a monospecialty orthopedic hospital, which may have restricted the continuity and integration of multidisciplinary diagnostic information. As a result, relevant data, particularly those related to the patient’s colorectal evaluation, may have been fragmented or delayed during inter-hospital communication and referral.

## 4. Conclusions

This case report supports the association between *S. bovis* bacteremia and underlying colorectal pathology. Although PJIs caused by this pathogen are rare, clinicians should maintain a high index of suspicion and urge for comprehensive gastrointestinal evaluation in such cases. Early diagnosis and intervention are critical to improving patient outcomes and ensuring successful management.

## Data Availability

The data presented in this study are available on reasonable request from the corresponding authors.

## Abbreviations

The following abbreviations are used in this manuscript:

CRP	C-reactive protein
ESR	Erythrocyte sedimentation rate
Hb	Hemoglobin
PJI	Periprosthetic joint infection
*S. bovis*	*Streptococcus bovis*
